# miR-127 Regulates Cell Proliferation and Senescence by Targeting BCL6

**DOI:** 10.1371/journal.pone.0080266

**Published:** 2013-11-25

**Authors:** Jingwen Chen, Miao Wang, Mingzhou Guo, Yuntao Xie, Yu-Sheng Cong

**Affiliations:** 1 Key Laboratory for Cell Proliferation and Regulation Biology of Ministry of Education, Institute of Cell Biology, College of Life Sciences, Beijing Normal University, Beijing, China; 2 Institute of Aging Research, Hangzhou Normal University School of Medicine, Hangzhou, China; 3 Department of Gastroenterology &Hepatology, Chinese PLA General Hospital, Beijing, China; 4 Breast Center, Beijing Cancer Hospital & Institute, Peking University Cancer Hospital, Beijing, P. R. China; University of Barcelona, Spain

## Abstract

Cellular senescence occurs as a response to extracellular and intracellular stresses and contributes to aging and age-related pathologies. Emerging evidence suggests that cellular senescence also acts as a potent tumor suppression mechanism that prevents the oncogenic transformation of primary human cells. Recent reports have indicated that miRNAsact as key modulators of cellular senescence by targeting critical regulators of the senescence pathways. We previously reported that miR-127 is up-regulated in senescent fibroblasts. In this report, we identified miR-127 as a novel regulator of cellular senescence that directly targets BCL6. We further showed that miR-127 is down-regulated in breast cancer tissuesand that this down-regulation is associated with up-regulation of BCL6. Over-expression of miR-127 or depletion of BCL6 inhibits breast cancer cell proliferation. Our data suggest that miR-127 may function as a tumor suppressor that modulates the oncogene BCL6.

## Introduction

Cellular senescence was originally described by Hayflick five decades agoas an irreversible proliferation arrest of normal somatic cells [1]. Cellular senescence occurs in culture and in vivo as a response to extracellular and intracellular stresses, including telomere dysfunction, DNA damage caused by radiation or chemicals, and oncogenic or mitogenic stimuli [2], [3]. Cellular senescence causes permanent cell cycle arrest and, thereby, acts as a potent tumor suppression mechanism that prevents the oncogenic transformation of primary human cells [2], [4]. Senescence is a defining feature of premalignant tumors, andsenescent cells do not exist in malignant tumors. The induction and maintenance of cellular senescence is largely dependent on either or both of the p53/p21 and p16^INK4a^/pRB tumor suppressor pathways [5]. Recent studies haveindicated that microRNAs regulate cellular senescence by targeting the key regulators of cellular senescence pathways [6].

MicroRNAs (miRNAs) are small noncoding RNAs that play an important role in a variety of biological processes by negatively regulating expression of specific target genes at the post-transcriptional level. miRNAs regulate a variety of target genes involved in multiple pathways and processes, such as development, apoptosis, proliferation, differentiation, transformation, and cellular senescence [7], [8].

Using microarray, we previously identified a set of miRNAs differentially expressed in proliferating versus senescent human fibroblasts. miR-127-3p is one of the miRNAs that was up-regulated in senescent WI-38 and IMR-90 cells [9]. miR-127-3p and miR-127-5p are two mature miRNAs that are processed from the same precursor miRNA; hereafter, miR-127-3p will be referred to as miR-127. miR-127 is located in chromosome region 14q32.2 and belongs to a cluster that includes miR-431, miR-433, miR-127, miR-432, and miR-136 [10]. miR-127 and miR-433 are transcribed from independent promoters in overlapping genomic regions,and expression of these two miRNAs is induced by estrogen related receptor gamma (ERRγ) and inhibited by small heterodimer partner (SHP), a unique orphan nuclear receptor and transcriptional repressor [11]–[13]. It was reported that miR-127targets proto-oncogene BCL6 [14]. miR-127 is expressed at its highest level during the late stage of fetal lung development and may thus play an important role in this process [15]. In addition, miR-127 has been shown to regulate BCL6-mediated expression of CDKN1A (p21). In rat liver cells, down-regulation of miR-127 promotes cell proliferation, while up-regulation of miR-127 inhibits proliferation [16]. These observations suggest important roles for miR-127 in cell proliferation, differentiation, and development.

Here, we show that miR-127 induces senescence in human fibroblasts and inhibits the proliferation of breast cancer cells by targeting the oncogene BCL6. Additionally, we found an inverse correlation of expression between BCL6 and miR-127 in primary breast tumors versus adjacent normal tissues. Our data suggest that miR-127 is a novel senescence-associated (SA)-miRNA that regulates cellular senescence.

## Results

### miR-127 Overexpression Induces Cellular Senescence in Human Fibroblasts

**Figure 1:**
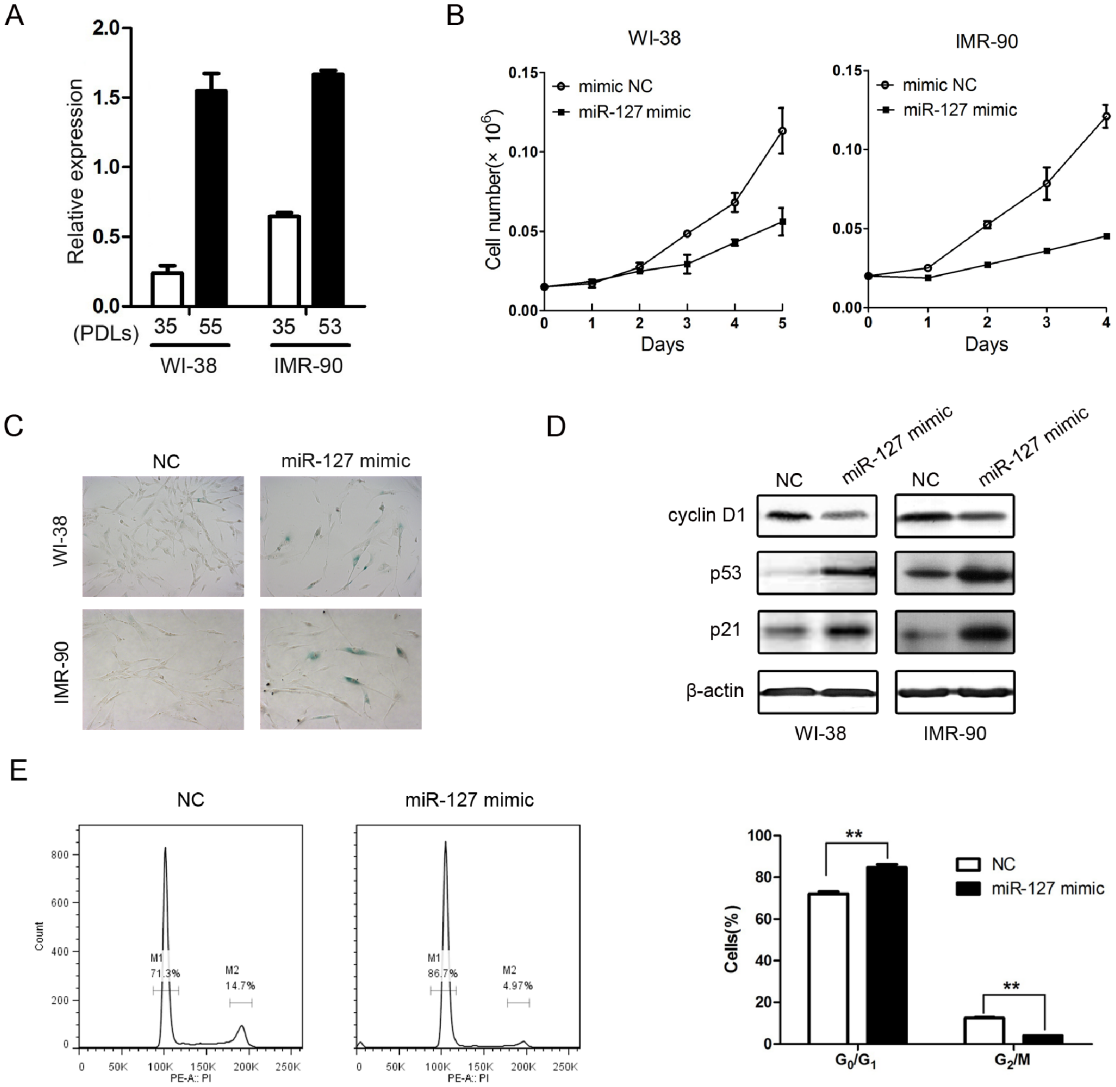
miR-127 is up-regulated in senescent human fibroblasts and mediates cellular senescence. (A) Relative levels of miR-127 expression analyzed by stem-loop qRT-PCR in different PDLs of WI-38 and IMR-90 cells. U6 RNA was used for normalization. Data are presented as the mean ± SD from three independent experiments. (B) Growth curves of WI-38 and IMR-90 cells transfected with 50 nM miR-127 mimic and negative control. Growth curves were generated by cell number at the indicated times. Data are presented as the mean ± SD from three independent experiments. (C) SA-β-gal staining of senescent cells induced by miR-127. The WI-38 and IMR-90 cellswere stained for SA-β-gal activity 7 days after transfection with 50 nM miR-127 mimic or negative control. (D) Western blot analysis of cyclin D1, p53, and p21 in WI-38 and IMR-90 cells at 72 hours post-transfection with the 50 nM miR-127-3p mimic or negative control. β-actin was used for normalization. (E) Cell cycle analysis was performed at 48 h after transfection. The histogram displays the percentage changes of G_0_/G_1_ and G_2_/M when WI-38 cells transfected with miR-127 mimics and negative control. M1 and M2 show the spike of G_0_/G_1_ and G_2_/M, respectively. Data are presented as the mean ± SD from three independent experiments (*P<0.05, **P<0.01).

**Figure 2:**
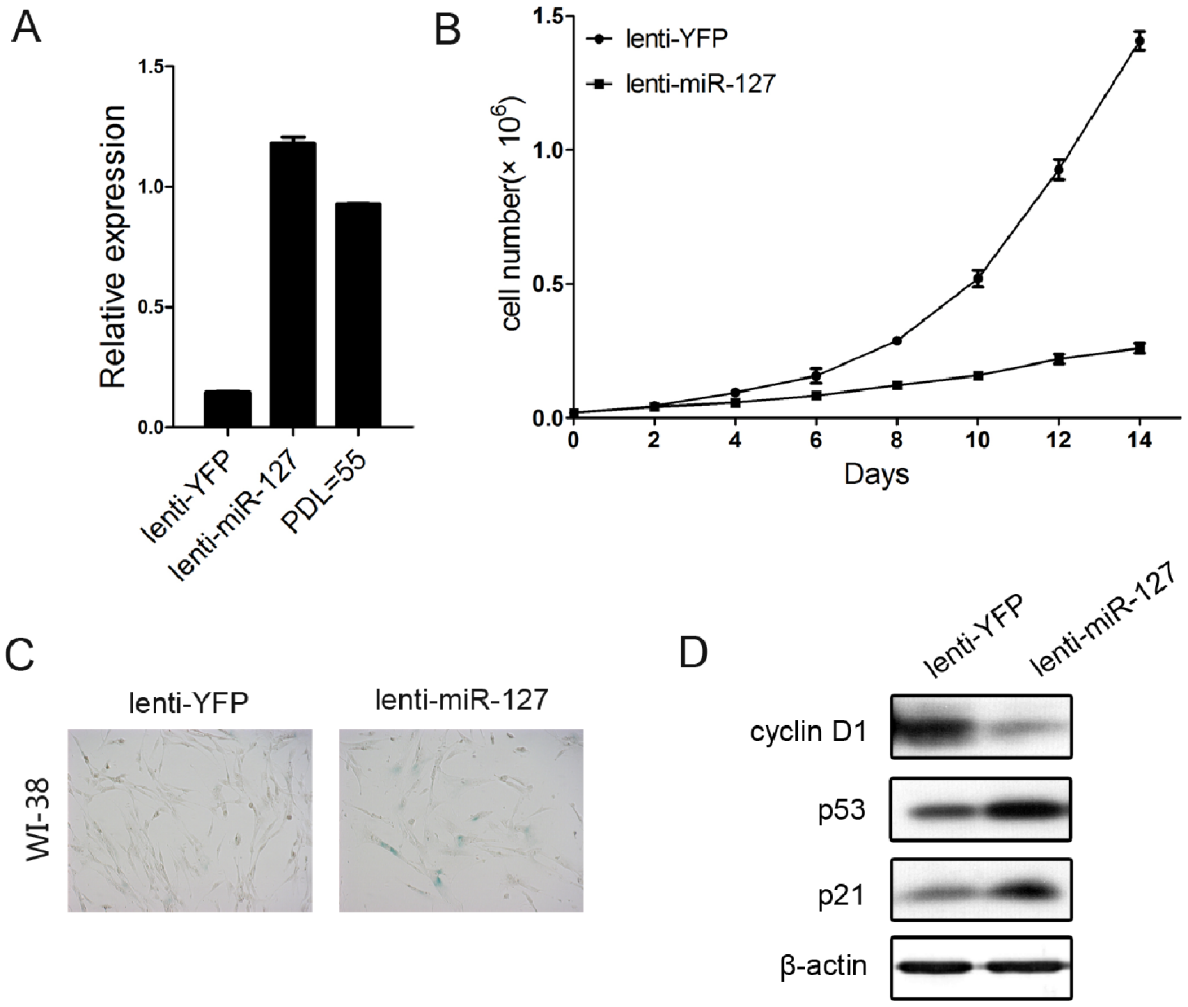
Stable expression miR-127 affects senescence phenotypes in WI-38 fibroblasts. (A) Stable expression of miR-127 in WI-38 cells infected by lentiviral constructs and endogenous expression of miR-127 in senescent WI-38 cells were evaluated by qRT-PCR analysis. Data are presented as the mean ± SD from three independent experiments. (B) Growth curves of WI-38 cells stably infected with pLenti6/V5-D-premiR-127 or pLenti6/V5-D-YFP. Cells were counted every two days for two weeks. Data are presented as the mean ± SD from three independent experiments. (C) SA-β-gal staining of senescent cells induced by stable overexpression of miR-127. The WI-38 cells were stained for SA-β-gal activity 10 days after lentiviral infection. (D) Western blot analysis of senescence-associated proteins in WI-38 cells 6 days after lentiviral infection. β-actin was used for normalization.

Using microarray, we previously reported that miR-127 is differentially expressed in young replicating versus senescent WI-38 cells [9]. To further confirm the microarray data, we performed real-time RT-PCR analysis on miR-127 in young proliferating and senescent WI-38 cells and IMR-90 cells. The results showed that miR-127 expression was up-regulated in senescent WI-38 cells and IMR-90 cells (Figure 1A). These findings suggest that miR-127 is a novel SA-miRNA. To investigate the involvement of miR-127 in cellular senescence in human fibroblasts, we induced miR-127 expression by transfecting a miR-127 duplex mimic into the young proliferating human fibroblast cell lines WI-38 and IMR-90. We observed that induced miR-127 expression caused a remarkable inhibition of cell proliferation (Figure 1B) and increased senescence-like phenotypes with positive staining of senescence-associated-β-galactosidase (SA-β-gal) (Figure 1C) in both WI-38 and IMR-90 cells. In addition, the senescence-like phenotype was associated withup-regulation of p53 and p21 and down-regulation of cyclin D1 (a pattern associated with senescence) in both WI-38 and IMR-90 cells (Figure 1D). As expected, miR-127 overexpression induced cell cycle arrest at G_0_/G_1_ phase (Figure 1E). This indicates that over-expression of miR-127 induces cellular senescence. To ensure that the observed effects of the miR-127 duplex mimic were not associated with supraphysiologic levels of miR-127 expression, we repeated these experiments using a lentiviral expression system. A pre-miR-127 lentiviral construct (lenti-miR-127) that stably expressesthe miR-127 precursor in its native context was used to study the effect of miR-127 on cellular senescence in WI-38 fibroblasts. We first confirmed that the level of miR-127 expressionwas up-regulated by the infection with lentiviral miR-127 and were comparable to that in senescent WI-38 (Figure 2A). Cells that were stably expressing miR-127 exhibited a reduced life span (Figure 2B), enlarged senescence morphology, and SA-β-gal positive staining (Figure 2C) compared with cells infected with the YFP lentivirus. Similar to the results of transient expression of miR-127, p53 and p21 were up-regulated, and cyclin D1 was down-regulated in WI-38 cells stably expressing miR-127. These results indicate that miR-127 indeed regulates cellular senescence in normal human fibroblasts.

### BCL6 is a Target of miR-127

**Figure 3:**
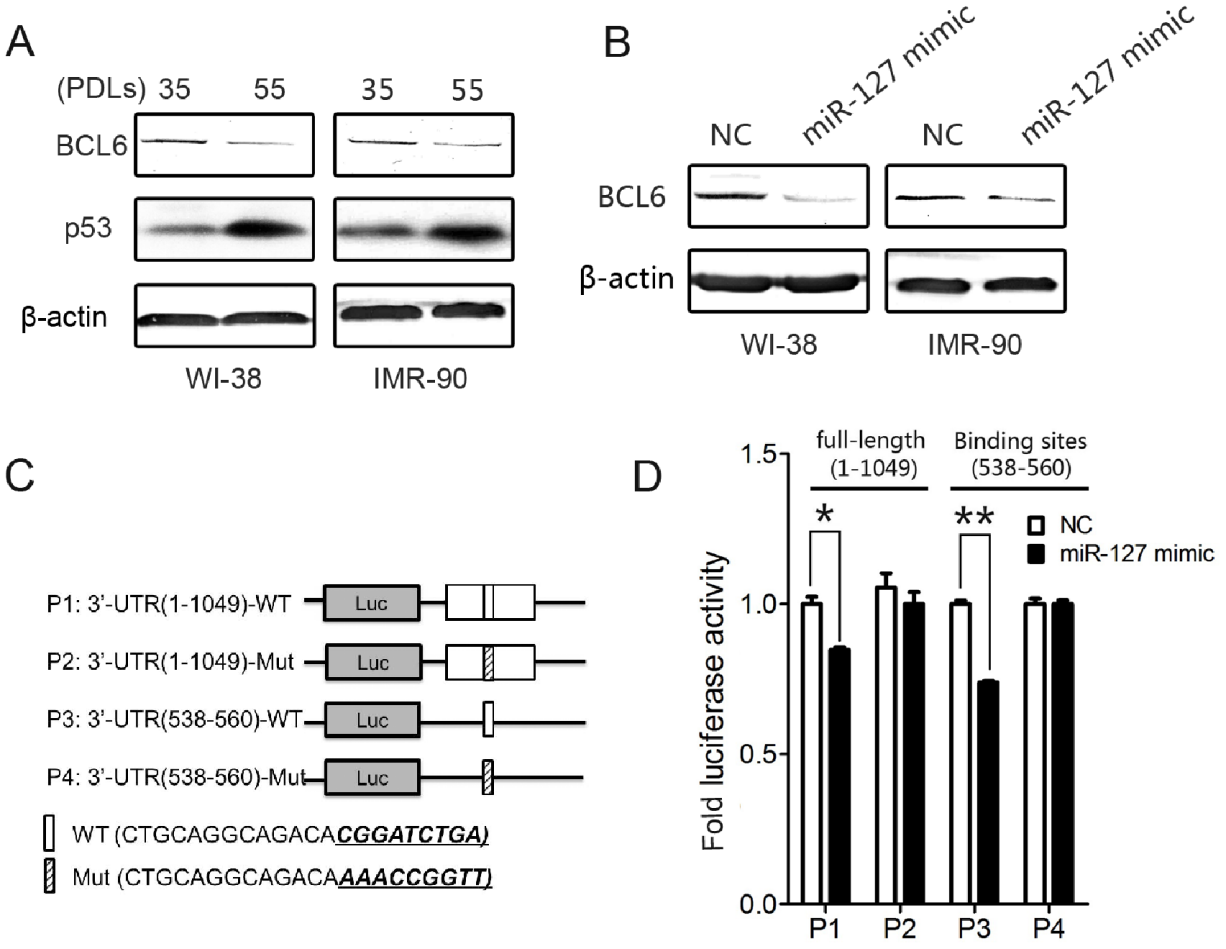
BCL6 is a direct target of miR-127. (A) Expression levels of BCL6 in different PDLs of WI-38 and IMR-90 cells. β-actin was used for normalization. (B) Representative Western blot analysis of BCL6 in WI-38 and IMR-90 cells transfected with 50 nM miR-127 mimic or negative control at 72 hours post-transfection. (C) Schematic representation of the luciferase reporter constructs containing full-length BCL6 3′-UTR (P1 contains wild-type miR-127 target sites and P2 is mutated in the miR-127 target sites) or the wild-type (P3) or mutant (P4) miR-127 target sites of BCL6 3′-UTR. (D) Luciferase reporter constructs (P1, P2, P3, and P4) along with pCMV-β-gal plasmid were cotransfected with miR-127 or negative control vector into MCF7 cells. Luciferase reporter assays were performed at 48 hours post-transfection. Data are presented as the mean ± SD from three independent experiments (*P<0.05, **P<0.01).

To investigate how miR-127 participates in the regulation of cellular senescence, we sought to identify miR-127 target genes in normal human fibroblasts. BCL6 have been reported to be a potential target of miR-127 [14]. Because miR-127 is significantly up-regulated in senescent cells, we first examined BCL6 expression in young and senescent fibroblasts by Western blot analysis. We found that, in inverse correlation with miR-127 expression, BCL6 was significantly down-regulatedin senescent WI-38 and IMR-90 fibroblasts (Figure 3A). Furthermore, over-expression of miR-127 has markedly down-regulated BCL6 expression in both WI-38 and IMR-90 cells (Figure 3B). To confirm that BCL6 is a direct target of miR-127 in human fibroblasts, we then performed luciferase reporter assays. We constructed luciferase reporters using the pGL3 vector that contained the wild-type full-length 3′-UTR ofBCL6 (Figure 3C, P1) orthe mutant full-length 3′-UTR of BCL6 (Figure 3C, P2), in which the miR-127 binding site was mutated by site-directed mutagenesis. Overexpression of miR-127 repressed the luciferase activity of the reporter containing the wild-type full-length 3′-UTR, but not the mutant full-length 3′-UTR (Figure 3D). In addition, we constructed luciferase reporters containing the wild-type (Figure 3C, P3) or mutant (Figure 3C, P4) binding site of miR-127 within the BCL6 3′-UTR. As is shown in Figure 3D, miR-127 significantly reduced the luciferase activity of the reporter containing the wild-type binding sitebut not the mutant binding site of miR-127. Together, these results suggest that BCL6 is a direct target of miR-127 in the context of senescence in human fibroblasts.

### BCL6 Regulates Cellular Senescence of Human Fibroblasts

**Figure 4:**
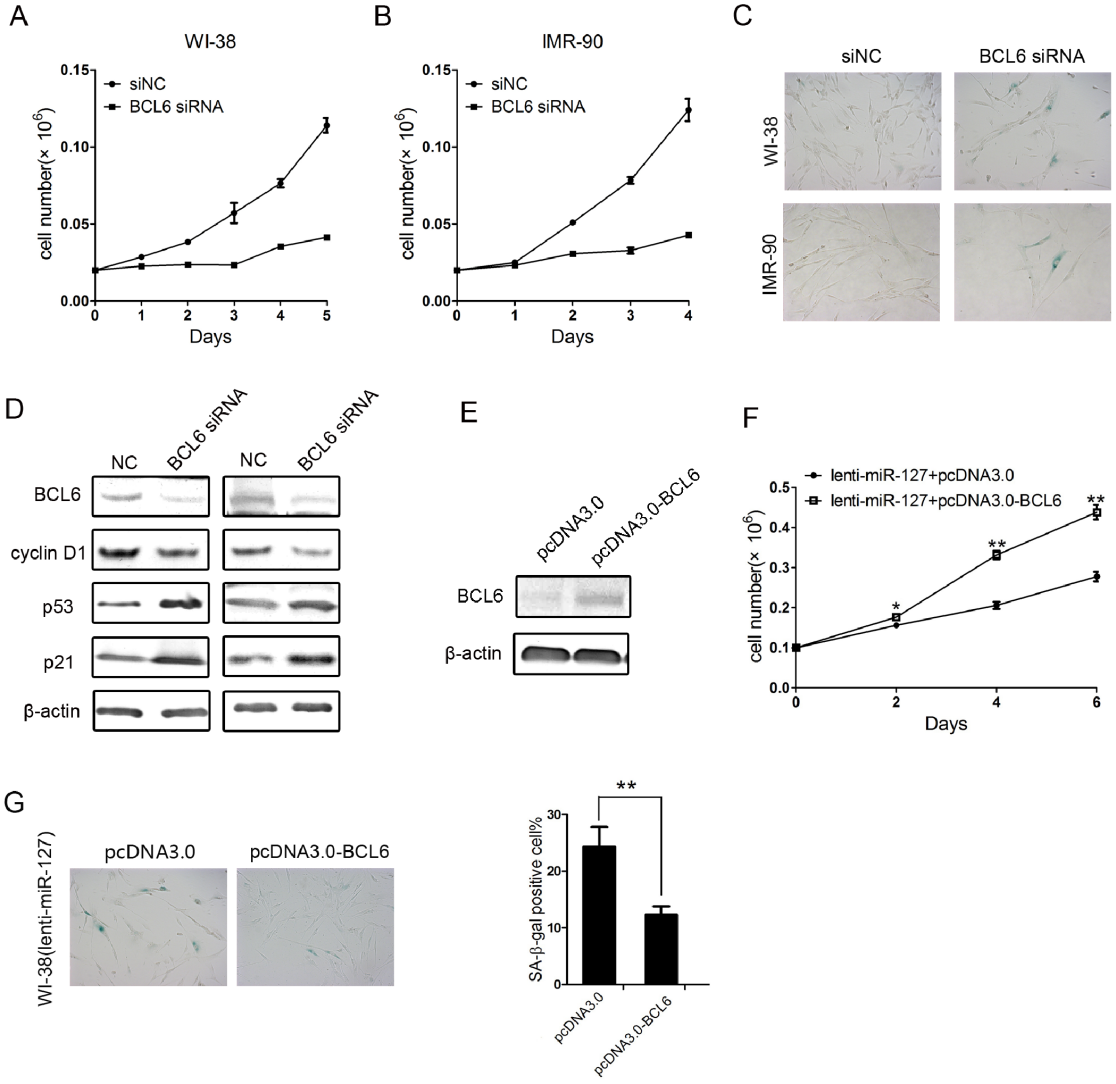
BCL6 is potentially involved in miR-127-mediated cellular senescence. (A–B) Growth curves of WI-38 and IMR-90 cells transiently transfected with 50 nM BCL6 siRNA or negative control. Growth curves were generated by cell number at indicated times. Data are presented as the mean ± SD from three-independent experiments. (C) SA-β-gal staining of senescent cells induced by transient transfection of BCL6 siRNA. The WI-38 and IMR-90 cells were stained 7 days after transfection with 50 nM BCL6 siRNA or negative control. (D) Western blot analysis of BCL6, cyclin D1, p53, and p21 in WI-38 and IMR-90 cells transiently transfected with 50 nM BCL6 siRNA or negative control. β-actin was used for normalization. (E) Western blot analysis of the expression of BCL6 in miR-127-expressing WI-38 cells transiently transfected with pcDNA3.0 and pcDNA3.0-BCL6. (F and G) Growth curve (F) and SA-β-gal activity (G) in miR-127-expressing WI-38 cells transfected by pcDNA3.0 and pcDNA3.0-BCL6. Cells were counted every two days for a week. The cells were stained SA-β-gal activity at 7 days after transfection with pcDNA3.0 or pcDNA3.0-BCL6. Data are presented as the mean ± SD from three independent experiments (*P<0.05, **P<0.01).

In mouse fibroblasts, it has been reported that BCL6 is a potent inhibitor of senescence that can bypass the senescence response downstream of p53 through a process that requires induction of cyclin D1 expression [17]. Therefore, we depleted BCL6 expression in WI-38 and IMR-90 human fibroblasts using ON-TARGET plus SMARTpool BCL6 siRNAs. As shown in Figure 4, the depletion of BCL6 inhibited cell proliferation (Figure 4A, B) and was associated with increased p53 and p21 expression and SA-β-gal-positive staining in WI-38 and IMR-90 fibroblasts (Figure 4C). We also observed that depletion of BCL6 resulted in down-regulation of cyclin D1 expression, which was previously confirmed to be a target of BCL6 [17]. This indicates that the depletion of BCL6 resulted in a similar senescence phenotype to miR-127 over-expression. Furthermore, we attempted to determine whether overexpression of BCL6 could rescue the senescence phenotype in WI-38 cells induced by overexpression of miR-127. We constructed the pcDNA3.0-BCL6 expression vector that lacks 3′-UTR of BCL6 gene. Our results showed that miR-127-induced senescence-associated phenotypes were rescued by the ectopically expressed BCL6 in WI-38 cells (Figure 4F,G). Our data suggest that miR-127 regulates cellular senescence through the p53/p21 pathway by targeting BCL6.

### miR-127 and its Target BCL6 Regulate the Proliferation of Breast Cancer Cells

**Figure 5:**
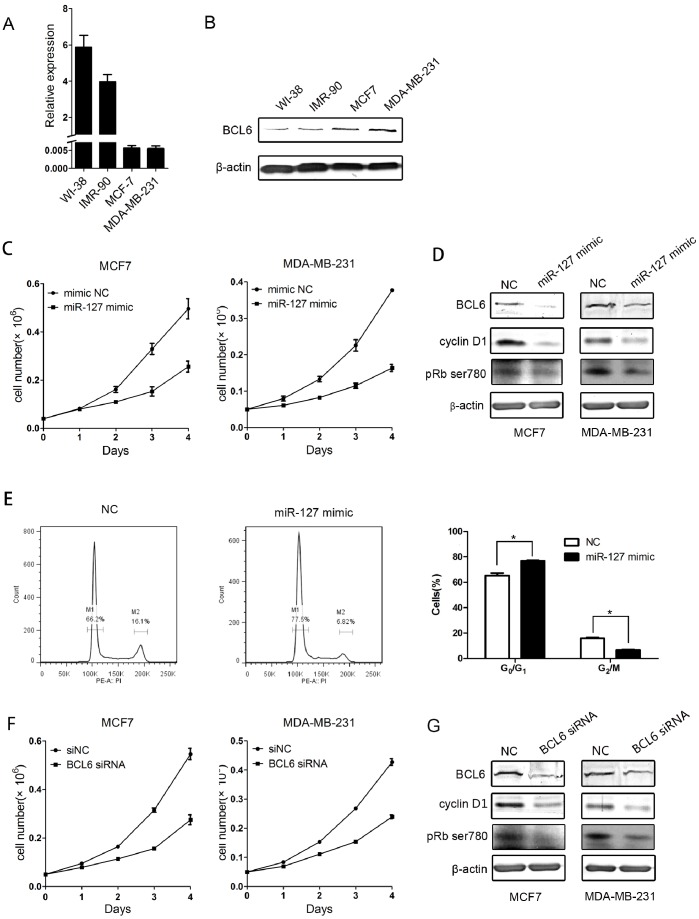
MiR-127 and BCL6 modulate the proliferation of breast cancer cell lines. (A) qRT-PCR analysis of miR-127 expression in WI-38, IMR-90, MCF7, and MDA-MB-231. U6 RNA was used for normalization. Data are presented as the mean ± SD from three independent experiments. (B) Western blot analysis of BCL6 in human fibroblasts, WI-38 and IMR-90, and the breast cancer cell lines MCF7 and MDA-MB-231. β-actin was used for normalization. (C) Growth curves of MCF7 and MDA-MB-231 cells transiently transfected with miR-127 mimic or negative control. Growth curves were generated by cell number at indicated times. Data are presented as the mean ± SD from three independent experiments. (D) Western blot analysis of BCL6, cyclin D1 and Phospho-Rb (Ser780) in MCF7 and MDA-MB-231 cells transiently transfected with the 50 nM miR-127-3p mimic or negative control. β-actin was used for normalization. (E) Cell cycle analysis was performed at 48 h after transfection.The histogram displays the percentage changes of G_0_/G_1_ and G_2_/M when MCF7 cells transfected with miR-127 mimics and negative control. M1 and M2 show the spike of G_0_/G_1_ and G_2_/M, respectively. (F) Growth curves of MCF7 and MDA-MB-231 cells transiently transfected by 50 nM BCL6 siRNA or negative control. (G) Western blot analysis of BCL6, cyclin D1 and Phospho-Rb (Ser780) in MCF7 and MDA-MB-231 cells 72 hours after transfection with 50 nM BCL6 siRNA or negative control. β-actin was used for normalization.

Accumulating evidence indicates that cellular senescence is a tumor suppression mechanism that prevents the oncogenic transformation of primary human cells [18]. We therefore examined the expression of miR-127 in breast cancer cells. The results showed that miR-127 has a much lower expression level in breast cancer MCF7 and MDA-MB-231 cell lines compared with normal human fibroblasts (Figure 5A). In contrast, BCL6 expression had a higher expression level in breast cancer cells than normal cells (Figure 5B). To further investigate the role of miR-127 in breast cancer cell proliferation, we transfected miR-127 mimics into the breast cancer cell lines MCF7 and MDA-MB-231. miR-127 significantly inhibited growth in both the MCF7 and MDA-MB-231 cell lines and was associated with down-regulation of BCL6, cyclin D1 and dephosphorylation of pRb at ser780 (Figure 5C, D). Rb protein (pRb) is responsible for a major G1 checkpoint, blocking S-phase entry and cell growth. We then analyzed the cell cycle of MCF7 cells transfected by miR-127 mimics. We observed an increased cell cycle arrested at G_0_/G_1_ (Figure 5E). This was consistent with the effects on cell cycle in normal fibroblast WI-38. Similarly, the depletion of BCL6 inhibited cellular proliferation in the MCF7 and MDA-MB-231cell lines and was associated with down-regulation of cyclin D1 and a decrease in pRB phosphorylation at ser780 (Figure 5F, G). Additionally, we have performed the rescue experiments in MCF7 and MDA-MB-231 breast cancer cell lines (see Figure S1). We found miR-127-induced cell growth repression can be rescued by the ectopically expression of BCL6 in these two cell lines. Together, these results suggest that miR-127 inhibits cell proliferation by targeting BCL6 in breast cancer cells.

### Reverse Correlation between miR-127 and BCL6 Expression in Primary Breast Tumors Versus Matched Adjacent Tissues

**Figure 6:**
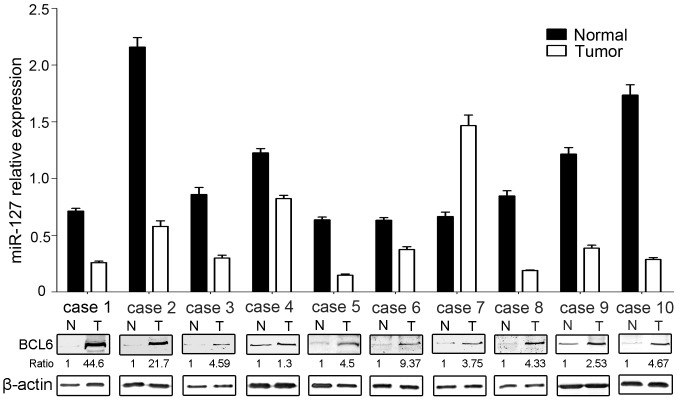
Expression levels of miR-127 and BCL6 in normal breast tissues and primary breast tumors. qRT-PCR analysis of miR-127 and Western blot analysis of BCL6 in matched sets of normal tissues and primary tumors of breast. U6 RNA was used for qRT-PCR normalization. Data are presented as the mean ± SD from three independent experiments. β-actin was used for Western blot normalization. N, normal tissues; T, corresponding tumors.

We then analyzed the expression of miR-127 in human breast cancer tissues and adjacent normal tissues. We found that miR-127 was down-regulated in 9 of 10 breast cancer tissues and conversely, that BCL6 expression was up-regulated in breast tumors compared with their corresponding tumor-adjacent tissues (Figure 6). In primary breast tumors,miR-127 expression is lower, and BCL6 expression is higher than in adjacent normal tissues, suggesting that miR-127 may function as a novel tumor suppressor and that its alteration may contribute to breast cancer development.

## Discussion

Cellular senescence is believed to be a tumor suppressor mechanism. Several microRNAs have been shown to target the oncogene and tumor suppressor networks, which have shared mechanisms in aging and cancer. Microarray studies have identified large subsets of miRNAs that are up-regulated or down-regulated in senescent cells, but only a few miRNAs have been shown to modulate senescence experimentally. miR-34a is a tumor suppressor that down-regulates a program of genes that promote cell cycle progression and isalso a potent inducer of cellular senescence [19]. miR-203 induces senescence by targeting the E2F3 protein in human melanoma cells [20]. Similarly, miR-22 targets CDK6, SIRT1, and Sp1, which are genes involved in the senescence program, and induces cellular senescence in both human fibroblast and breast cancer cells [21]. Little is known about the role of miR-127 in cellular senescence. In this report, we identified miR-127 as a novel regulator of cellular senescence that targets BCL6. miR-127 is up-regulated in senescent fibroblasts, and its over-expression inhibits proliferation and induces senescence-like phenotypes, including increased SA-β-gal-positive staining and up-regulation of p53 and p21 expression. This indicates that miR-127 mediates cellular senescence by activating the p53/p21 pathway in human fibroblasts. Our results also showed that BCL6 is a direct target of miR-127 in normal human fibroblasts. BCL6 is known to repress p53 transcription by binding two specific DNA sites within the p53 promoter region [22], [23]. This suggests that up-regulation of miR-127 in senescent cells can activate p53 by translational repression of BCL6. Interestingly, we observed that miR-127 and its target, BCL-6,are inversely correlated and that their expression profiles are opposite in breast tumors and matched adjacent tissues. In addition, we found an opposing effect of miR-127 and its target,BCL6, on breast cancer cell proliferation.

In both replicative senescence, caused by telomere shortening, and premature senescence, induced by oncogenes or oxidative stress, the DNA damage response is acommon mechanism for initiation of cellular senescence [3], [5], [24], [25]. miR-127 is up-regulated in replicative senescent cellsand in response to DNA damage induced by the DNA damage agent etoposide in WI-38 cells (data not shown). This suggests that miR-127 is involved in the DNA damage response by indirectly targeting p53 through the down-regulation of BCL6. The DNA damage response is a candidate anti-cancer barrier in early human tumorigenesis [26]. It has been reported that miR-127 is significantly reduced in gastric cancer tissues and osteosarcoma cell lines [27] and that BCL6 is overexpressed in invasive breast cancers [28], [29]. In breast cancer tissues, we found that miR-127 was down-regulated, whereas its target,BCL6, was up-regulated. Moreover, we showed that over-expression of miR-127 or depletion of BCL6 expression inhibited breast cancer cell growth. Together, these observations suggest that miR-127 functions as a novel tumor suppressor that coordinates with oncogene BCL6 to contribute to the pathologies of aging and cancer.

## Materials and Methods

### Cell Culture

The WI-38, IMR-90, MCF7 and MDA-MB-231 cell lines were obtained from ATCC. WI-38, IMR-90, and MCF7cells were grown in Dulbecco’s modified Eagle’s medium. MDA-MB-231 was cultured in RPMI1640 medium. All media were supplemented with 10% FBS (vol/vol), 1% L-glutamine, 100 U/ml penicillin, and 100 µg/ml streptomycin.

### Transient miRNA/siRNA Transfection and Plasmid Transfection

The hsa-miR-127 mimic and negative controls were obtained from GenePharma (Shanghai, China). BCL6 siRNA was purchased from Thermo Scientific (ON-TARGET plus SMARTpool Human BCL6; Waltham, MA, USA). The sequences of the siRNAs against BCL6 were as follows: 5′-CCUUAAUCGUCUCCGGAGU-3′, 5′-GUAUAUACCCGUACAACGU-3′, 5′-GUUAUAACUACUCCGGAGA-3′, and 5′-CAUCAAGCCUCCUCGUGAA-3′. The cells were transfected with 50 nM of either miRNA or siRNA using Lipofectamine^TM^2000 (Invitrogen, Carlsbad, CA, USA) according to the manufacturer’s protocol. The transfection efficiency under the conditions adopted in this study was estimated to be >90% by a fluorescence-labeled double-stranded siRNA.

For rescue experiments, the expression plasmid pcDNA3.0-BCL6 was used, which contained the encoding region of BCL6 mRNA but lack of the 3′-UTR. Cells stably infected by miR-127 lentivirus were transfected with 6 µg of pcDNA3.0-BCL6 in a 100-mm dish using Lipofectamine2000(Invitrogen) according to the manufacturer’s protocol for 24 h, and subsequently cells were calculated for every two days.10^4^ cells were seeded to 35-mm-diameter dishes and incubated for 5 days. The overexpression of BCL6 was confirmed by Western blotting.

### Lentivirus Infection

Lentiviruses were generated by co-transfecting the lentiviral vector (pLenti6/V5-D-YFP, pLenti6/V5-D-premiR-127) and packaging plasmid mix (7∶3∶4 for pLP1, pLP2, and pLP/VSVG) into 293T cells using Lipofectamine™ 2000 (Invitrogen, Carlsbad, CA, USA). Supernatants were collected 48 hours after transfection, filtered through a 0.22-µm membrane, and directly used to infect cells.

### Luciferase Reporter Assay

The BCL6 3′UTR segment was amplified by PCR with specific primers and subsequently cloned into the*Xba*I site of the pGL3-Control vector (Promega, Madison, WI, USA). The mutant BCL6 3′UTR segment, with point substitutions in the miR-127 complementary sites, was generated by site-directed mutagenesis. Oligonucleotides containing the wild-type or mutant target site of the BCL6 3′UTR were also cloned into the *Xba*I site of the pGL3-control vector (Promega, Madison, WI, USA). All the inserts were confirmed by sequencing. The reporter plasmids and pCMV-β-gal plasmid for normalization of transfection were cotransfected with miRNA duplex in a 24-well plate using Lipofectamine™ 2000 (Invitrogen, Carlsbad, CA, USA). The cells were then incubated for 48 hours, and luciferase activity was determined using the luciferase assay system (Promega, Madison, WI, USA). β-gal activity was determined with the β-galactosidase enzyme substrate ONPG (Sigma-Aldrich, St. Louis, MO, USA). Luciferase activity was then normalized with respect to β-gal activity.

### Quantitative Real-time RT-PCR

Total RNA was prepared from cell lines using theTRIzol reagent (Invitrogen, Carlsbad, CA, USA) according to the manufacturer’s instructions. RNAs were quantified by measuring UV absorbance at 260 nm, and their quality was assessed by 1.2% agarose/formaldehyde gel electrophoresis under denaturing conditions. miRNA expression levels were detected by real-time RT-PCR following a previously described protocol [30]. Total RNAs were transcribed by M-MLV reverse transcriptase (Promega, Madison, WI, USA) using the stem-loop reverse primers. Real-timePCR using SYBR Green Supermix (Bio-Rad, Hercules, CA, USA) was performedwith an iQTM5 multicolor real-time PCR detection system (Bio-Rad, Hercules, CA, USA). The PCR conditions were as follows: 3 minat 95°C, followed by 40 cycles of 95°C for 15 s, 60°Cfor30 s, and 72°C for 30 s. U6 was measured as an internal control. The primer sequences used for the real-time RT-PCR experiments are available upon request.

### Cell Cycle Analysis

48 h after transfection, cells were fixed in 70% ice-cold ethanol and stored at −20°C overnight. The cells were then washed twice with phosphate buffered saline (PBS) and resuspended in 0.5 ml PBS containing 100 µg/ml RNase A. After RNase treatment at 37°C for 30 minutes, the samples were stained with PI and analyzed using LSRFortessa cytometer (BD Biosciences). TheG_0_/G_1_ and G_2_/M ratios were calculated by using analysis software (FlowJo).

### SA-β-gal Staining

Cell staining for SA-β-gal activity was performed as described in a previous study [31]. In brief, the cells were washed with PBS, fixed in 2% formaldehyde and 0.2% glutaraldehyde, washed, and incubated at 37°C for 12–16 hours with SA-β-gal staining solution.

### Immunoblot Analysis

Cells were homogenizedin RIPA lysis buffer (50 mMTris/HCl pH8, 150 mMNaCl, 1% NP-40, 0.5% sodium deoxycholate, and 0.1% SDS) with a protease inhibitor cocktail (Roche Diagnostics, Mannheim, Germany). Thirty micrograms of total cell lysateswere separated by SDS-PAGE and transferred to polyvinylidene fluoride membranes. Antibodies top53 (Cell Signaling Technology, Beverly, MA, USA), p21 (Cell Signaling Technology, Beverly, MA, USA), BCL6 (Cell Signaling Technology, Beverly, MA, USA), Cyclin D1 (Cell Signaling Technology, Beverly, MA, USA), Phospho-Rb (Ser780) (Cell Signaling Technology, Beverly, MA, USA) and β-actin (Beijing Zhongshan Biotechnology, Beijing, China) were purchased.

### Primary Tissue Samples

Matched sets of primary breast tumors and adjacent normal tissues from the same patients were obtained after patients signed informed consent forms and the studies were approved by the Medical Ethics Committee of the Breast Center, Peking University Cancer Hospital.

### Statistical Analysis

The samples for cell growth, quantitative RT-PCR analysis and promoter luciferase assays were run in triplicate, and the experiments were repeated at least three times. Data were analyzed by a Student’s t-test to determine statistical significance. p<0.05 was considered significant. *represents a p-value <0.05, **p-value <0.01.

## Supporting Information

Figure S1 **MCF7(A) and MDA-MB-231 (B) cells were first transfected with miR-127 duplex.** 24 h after transfection, cells were subsequently transfected with pcDNA3.0 and pcDNA3.0-BCL6, respectively. Cells were counted everyday for 4 days. Data are presented as the mean ± SD from three independent experiments (*P<0.05, **P<0.01).
(TIF)
