# Supplementary figures and images for: miR-127 Regulates Cell Proliferation and Senescence by Targeting BCL6
Source: PLoS One. 2013 Nov 25;8(11):e80266. doi: 10.1371/journal.pone.0080266 (PMC3840165; doi:10.1371/journal.pone.0080266)

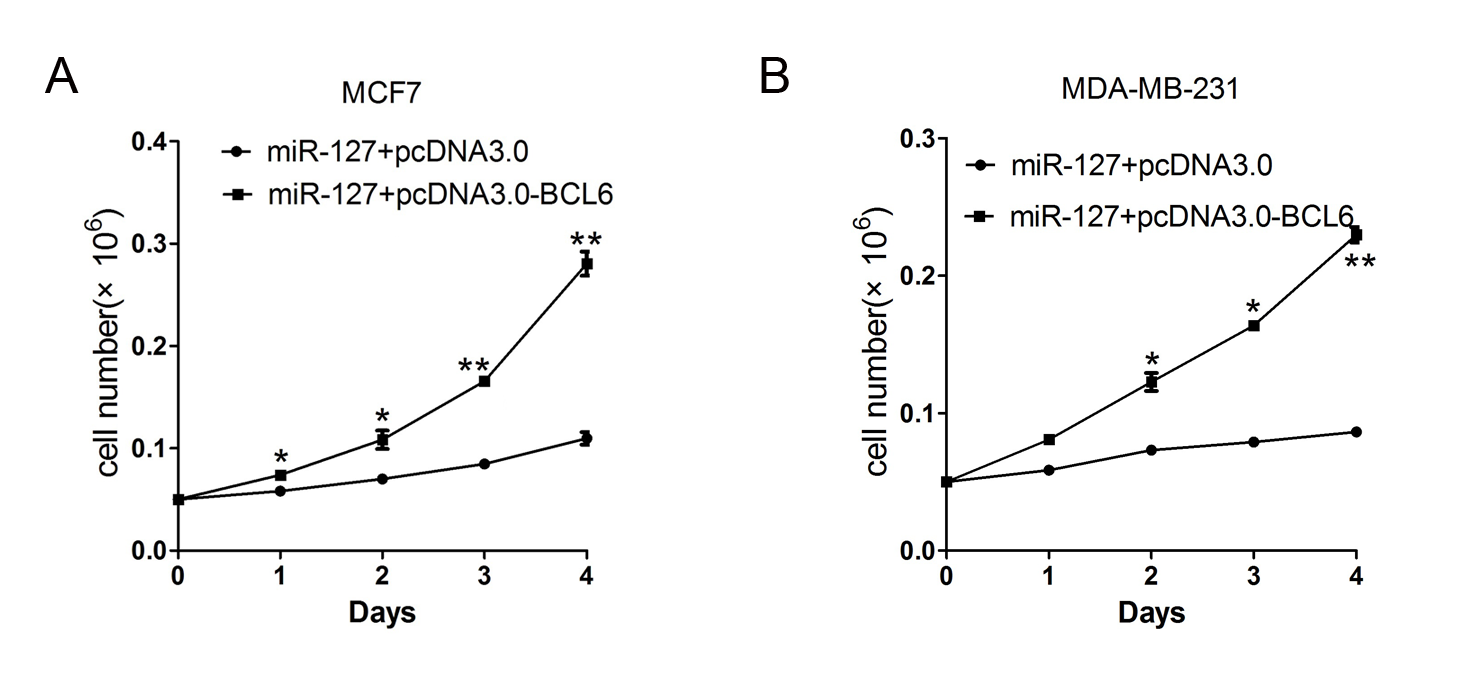

Supplement: Figure S1 — MCF7(A) and MDA-MB-231 (B) cells were first transfected with miR-127 duplex. 24 h after transfection, cells were subsequently transfected with pcDNA3.0 and pcDNA3.0-BCL6, respectively. Cells were counted everyday for 4 days. Data are presented as the mean ± SD from three independent experiments (*P<0.05, **P<0.01). (TIF) [file pone.0080266.s001.tif]
